# Supplementary material for: Effects of amotosalen treatment on human platelet lysate bioactivity: A proof-of-concept study
Source: PLoS One. 2020 Apr 15;15(4):e0220163. doi: 10.1371/journal.pone.0220163 (PMC7159197; doi:10.1371/journal.pone.0220163)
Supplement: Attachment — Submitted filename: Response to reviewers.docx [file pone.0220163.s001.docx]

**Below are responses to the reviewer’s comments**

**Reviewer 1**:

**Comment 1**: *The very large ranges in the % differences in the growth factors between the two batches of PIPC and PIPL makes it hard to know what a representative ‘baths’ of these hPL really are. I believe to really make any comment on the concentrations of growth factors in this setting, and to really interpret the results, more than 2 batches must be compared. Not only are there differences between the two hPL pathogen inactivation techniques, but also between the batches of the same technique…. This is a problem for interpreting the results.*

**Response**: We thank the reviewer for pointing this out. We agree with this comment and a line has been included to clarify that the individual difference of the two batches and the implication on the ability to generalize is a limitation of the study (page 12, line 213). It would indeed have been interesting to assess the concentrations of growth factors using a larger pool of batches, however for this proof-of-concept study we were only able to produce platelet concentrates from a total of 32 buffy coats. We have taken this into account for future studies.

**Comment 2**: *It is disappointing that there is not a control with the standard fetal bovine serum. Given the literature suggests that conventional pathogen inactivated hPL is as good or better than standard fetal bovine serum, it would be important to compare this method to the conventional AND post-lysate pathogen inactivation methods. This is especially important given the concentrations of selected soluble growth factors were lower in the post-lysate pathogen inactivation group, because it is important to know if these concentrations are of equivalence to the standard bovine fetal serum (as there may be a ceiling effect in the amount of growth factor needed for proliferation and differentiation, further supporting less importance on the growth factor differences and more importance on the biologic effects).*

**Response**: We thank the reviewer for addressing this. This is a valid point and we fully agree that a comparison to fetal bovine serum is valuable. However, for this proof-of-concept study where the central focus was to assess whether it was possible to produce platelet lysates by performing pathogen inactivation post-expiry, we believe that including fetal bovine serum would be slightly out of scope. The direct comparison between hPL and fetal bovine serum has been carried out in several papers including two by our own research group. See:

<https://www.ncbi.nlm.nih.gov/pmc/articles/PMC5506010/>
<https://www.ncbi.nlm.nih.gov/pubmed/25198449>
<https://www.ncbi.nlm.nih.gov/pubmed/23874839>

**Comment 3**: *Along these lines, the study is missing a limitations section that should be added, and should include discussion of the important issues with changing from a non-human source to a human blood-product source need to be mentioned as the pathogen reduction techniques may not completely mitigate the risk of viral transmissions.*

**Response**: We agree and a line in the introduction has been added accordingly, clarifying that the INTERCEPT™ Blood System does not guarantee 100% pathogen reduction and is indeed bypassed by non-enveloped viruses such as hepatitis A and hepatitis E (page 4, line 54).

**Comment 4**: *The interpretation of the results should be tempered. Given there were lower concentrations of key growth factors, lower cumulative population doublings, no comparison to standard fetal bovine serum, and no study of retention of genomic stability of avoidance of tumorigenicity, it is unclear to me whether this successfully supports long term cell proliferation.*

**Response**: We agree with the reviewer that the results in the submitted state did not fully support the conclusion that hPL-PIPL supports long-term cell proliferation alone. We refrained from the wording “successful” and added a line in the discussion pointing out to fully elucidate the effect on long-term cell proliferation, assessment of genomic stability and tumorigenicity is important (page 13, line 225). We also added to the conclusion to clarify that hPL-PIPL is solely viewed as a results of a possible production method and may be a valuable tool moving forward, but the results do not support the notion that hPL-PIPL is better or worse than conventional hPL-PIPC. Further studies are needed to follow up on this.

**Comment 5**: *Please discuss the decision to store for 3 weeks of -80 degrees. Why was this chosen? The LOS at -80 may effect results and needs to be studied going forward.*

**Response**: This is a very important point. We do not have reason to believe that this had a negative impact on the quality of the end product as it has been discussed as general practice by several groups (<https://www.ncbi.nlm.nih.gov/pubmed/29071726>). For this study, we decided upon these storage conditions due to practical reasons. By the time the platelet concentrates were ready to be processed by the National Blood Bank, we were awaiting the arrival of the first donor cells. Optimally, we would have preferred the avoid storage at all, but it was not possible.

**Comment 6**: *Please spell out the growth factors in the manuscript and in the table, they cannot be assumed to be known.*

**Response**: We thank the reviewer for this suggestion. We have spelled out the growth factors in the manuscript.

**Reviewer 2**:

**Comment 1**: *All the experiments were done using fresh platelets, but authors let the platelets expire. We think that experiments using the banked expired platelets should be performed to complement current findings.*

**Response**: We thank the reviewer for this comment. It is important for us to stress that the platelet concentrates used in the study were all banked and allowed to expire prior to experimentation. We have previously compared fresh platelets to expired platelets (<https://www.ncbi.nlm.nih.gov/pubmed/23874839>) but this was out of the scope of this study. Rather we wanted to study the possibility of producing viable platelet lysates from expired platelet concentrates applying pathogen inactivation post-expiry. We hope this answers the reviewer’s concern.

**Comment 2**: *In every section of the results, author should provide the rationale for each of the experiments, what they did, explain the results and their inferences, as much as possible and a brief manner.*

**Response**: We agree with this comment. However, we believe that the rationales of the experiments are well-described in the discussion section of the paper. E.g. at page 11, line 199 we argue that growth factors within the α-granules are important for MSC proliferation and differentiation and provided the basis for quantifying soluble growth factors and cytokines. At page 12, line 216 we discuss the impact of “higher” concentrations of growth factors on MSC proliferation supporting the use of the cumulative population doubling assay. Similar rationale is provided for each of the differentiation assays throughout the discussion.

**Comment 3**: In equations 1 and 3, authors should “write” multiplication symbol instead of using a “dot”.

**Response**: We thank the reviewer for this suggestion. We have replaced the use of a “dot” with the multiplication symbol “x” in equation 1 and 3 at page 16, line 311 and page 20, line 401.

**Comment** 4: In every place authors should correct CO2/CO2 (should be CO2).

**Response**: We thank the reviewer for this observation. It is correct that we falsely used the term “CO^2^” instead of the correct form “CO_2_”. This has been corrected at all places.
