# Supplementary material for: Effects of amotosalen treatment on human platelet lysate bioactivity: A proof-of-concept study
Source: PLoS One. 2020 Apr 15;15(4):e0220163. doi: 10.1371/journal.pone.0220163 (PMC7159197; doi:10.1371/journal.pone.0220163)
Supplement: Attachment — Submitted filename: Response to reviewers_OES.docx [file pone.0220163.s002.docx]

**Below are responses to the reviewer’s comments on the revised manuscript**

**Reviewer 1**:

**Comment 1**: *Unfortunately, based on my prior review, very few changes have been made in the manuscript (only a few short sentences added and a couple of words changed), and their responses do not adequately address my concerns*

**Response**: Based on this and Reviewer 1’s further comments on the revised manuscript, we have further expanded upon the concerns raised. Further details on specific issues are described below.

**Comment 2**: *They have now called this study a ‘proof-of-concept’ study in the reviewer responses, (but this is mentioned only once in their discussion). I would suggest, that if this is indeed a ‘proof-of-concept’ study, this be clarified throughout the ENTIRE manuscript including adding those words to the title so this is not misleading to readers*

**Response**: Thank you for pointing out that we needed to clarify that this is a “proof-of-concept” study throughout. As suggested, we have reworked the title and it now specifically references that this is a proof-of-concept work (page 1). We have also made sure to make this clear in the abstract (page 2, line 27), in the introduction (page 4, line 95), in multiple places in the discussion section (page 12, line 205; page 12, line 210; page 13 line 232; page 14, line 271), and in the conclusion (page 15, line 285).

**Comment 3**: *. As previously mentioned, the findings are not interpretable without more than two batches due to the very large ranges in the % differences in the growth factors between the two batches of PIPC and PIPL (not only are there differences between the two hPL pathogen inactivation techniques, but also between the batches of the same technique), and the missing bovine fetal serum comparison group (given the citations of direct comparisons performed between hPL and bovine fetal serum by this author group, there should be no reason why this cannot be performed for this study)..*

**Response**: This large differences between the two batches does indeed make it difficult to generalize the results of this study to hPL in general. We believe that, as this was a proof-of-concept study (unfortunately we did not make this clear in the original submission), that our results provide enough evidence to suggest that the application of pathogen inactivation after platelet lysis is worth further study. This has now been more explicitly stated in the manuscript (page 14, lines 274-282), and we have also clarified the limitations resulting from the small number of replicates (pages 12-13, lines 232-234; page 14, lines 271-274)

With regard to a comparison with FBS, we have added to the discussion a note that it was not included in this work because previous studies have shown hPL to be comparable to FBS as a cell culture supplement (page 12, lines 209-211). We have also noted that future work should include an FBS control (page 14, lines 279-280). Unfortunately, none of the studies references specifically looked at growth factor and cytokine levels in the FBS supplements used, so we cannot provide a comparison within our manuscript to previous results. We don’t believe that it is warranted to analyze the levels of these factors in FBS at this point, considering the study is already complete, but, again, we have noted that this should be included in future work.

**Comment 4**: *). The couple short sentences added to the manuscript without a focus on a full limitations section (as previously suggested), will not suffice given the significant limitations in current form. Given the many limitations, these should be addressed together in a true limitations section.*

**Response**: In order to address this concern, in addition to the sentences previously added into the manuscript after the initial review, we have added a paragraph at the end of the discussion section which discusses how the limitations inherent in this proof-of-concept work should be addressed in future work. Please note that the main limitations are explicitly stated within this discussion. (page 14, lines 274-282)

**Comment 5**: *[The limitations] need to include the issue raised of storage for 3 weeks at -80 especially given it was decided based on practical reasons. (comment base on the initial submission:* *Please discuss the decision to store for 3 weeks of -80 degrees. Why was this chosen? The LOS at -80 may effect results and needs to be studied going forward.)*

**Response**: Again, thank you for raising this important point. As we initially stated, we do not have reason to believe that this had a negative impact on the quality of the end product as it has been discussed as general practice by several groups (<https://www.ncbi.nlm.nih.gov/pubmed/29071726>). For this study, we decided upon these storage conditions due to practical reasons: by the time the platelet concentrates were ready to be processed by the National Blood Bank, we were awaiting the arrival of the first donor cells. Optimally, we would have preferred to avoid storage at all, but it was not possible.

We would also like to point out that the production of platelet lysates does involve temperature cycling from -80°C to 37°C three times, as stated in the Materials and methods section (lines 321-322). Therefore, it is not unreasonable to store the cells at -80°C, given that this temperature is used for lysis. In addition, it must be noted that even in the absence of any storage period, the cells would have been exposed to -80°C during the process of lysis, and therefore we do not have any reason to believe that the relatively brief storage period used would have had any detrimental effect on the platelets.

Finally, it is worth noting that both treatments were exposed to the -80°C storage conditions.
